# Hepatitis B vaccination status and associated factors among students of medicine and health sciences in Wolkite University, Southwest Ethiopia: A cross-sectional study

**DOI:** 10.1371/journal.pone.0257621

**Published:** 2021-09-21

**Authors:** Kassahun Haile, Abebe Timerga, Ayenew Mose, Zebene Mekonnen

**Affiliations:** 1 Department of Medical Laboratory Science, College of Medicine and Health Sciences, Wolkite University, Wolkite, Ethiopia; 2 Department of Biomedical Science, College of Medicine and Health Sciences, Wolkite University, Wolkite, Ethiopia; 3 Department of Midwifery, College of Medicine and Health Sciences, Wolkite University, Wolkite, Ethiopia; 4 Department of Nursing, College of Medicine and Health Sciences, Wolkite University, Wolkite, Ethiopia; China University of Mining and Technology, CHINA

## Abstract

**Background:**

Hepatitis B virus (HBV) infection is a significant global public health problem. Health care providers and medical students in developing countries including Ethiopia are at an increased risk of contracting HBV due to the high burden of this infection. The most effective way of prevention against HBV infection is vaccination of health care providers. However, there is a paucity of data on the HBV vaccination coverage among students of health science in Ethiopia. Therefore, this study aimed to determine HBV vaccination coverage and associated factors, level of knowledge, attitudes, and practices (KAP) towards HBV among students of medicine and health science at Wolkite University.

**Materials and methods:**

A cross-sectional study was conducted at Wolkite University among 417 study participants from November to December 2020. The study participants were recruited by using a simple random sampling technique. Data were collected using a self-administered structured questionnaire and analyzed using SPSS version 21. A binary logistic regression model was used to determine the factors associated with full-dose vaccination status. Statistical significance was set at P-value <0.05.

**Results:**

Out of the 417 study participants, 5.8% (95%CI: 3.8–7.9) received a full-dose of the HBV vaccine in this study. Unavailability and high cost of the vaccine were frequently mentioned reasons for not being vaccinated against HBV. About 73.6%, 36.2%, and 47% of participants had good knowledge, positive attitudes, and good practices towards HBV, respectively. Being male gender (AOR: 8.8; 95%CI: 2.9–27), rural residence (AOR: 3.6; 95%CI:1.2–10.6), positive attitude (AOR: 0.44; 95%CI: 0.1–1.1), good practice (AOR: 0.17; 95%CI: 0.05–0.5), medicine department (AOR: 5.9; 95%CI: 1.2–29), being second-year student (AOR: 11.7; 95%CI: 2.7–50.9), third-year student (AOR: 19; 95%CI: 4.25–45), and fourth-year student (AOR: 27; 95%CI: 5.8–56) were significantly associated factors with full-dose vaccination status.

**Conclusion:**

Our study revealed that only small proportions (5.8%) of study participants received full-dose HBV vaccination. Vaccinations of students before starting clinical attachments, provisions of training for students on infection prevention mechanism and universal precautions particularly on HBV, increasing the uptake of the HBV vaccine, creating awareness on attitude and practice of students towards HBV to enhance uptake of the vaccine are recommended.

## Introduction

Hepatitis B virus infection is a global public health problem, a study indicated 3.61% of the overall burden of HBV infection globally with a higher burden reported from the African (8.8%) and western pacific region (5.26%) [1]. Hepatitis B infection is a major public health concern in Ethiopia, a study reported 6%-9% burden of the infection [2, 3] and it is the most commonly transmitted blood-borne pathogen in healthcare settings [3]. Health care providers and medical students are at an increased risk of contracting HBV in the workplace due to occupational exposure to blood and body fluid during their clinical attachments [4]. Different epidemiological studies indicated 4.6%, 11%, and 9% the overall prevalence of HBV among medical and health science students at the University of Lome, Togo [5], Makerere University, Uganda [6], and Ethiopia [2], respectively. Some of the major reasons for the high prevalence of HBV among the students are lack of knowledge about HBV, non-adherence to standard precautions, and failure to take vaccine [4, 7, 8].

Transmission of HBV in a health care setting occurs through contact with blood, blood products, body fluids, needlestick, and sharps injury from HBV carrier [9, 10]. Medical and health science students as part of the healthcare delivery system they are exposed to the same size of risk as other health care workers when they come in contact with patients and contaminated instruments during their professional practice. A study conducted among nursing and midwifery students in Ethiopia at Haramaya and Jigjiga University showed 62.8% burden of needle stick and sharps injury [8]. Thus, planning, developing, and applying prevention mechanisms to reduce the risk of nosocomial infection among students was needed before starting their professional attachment in the health facility.

The most effective and efficient way of prevention mechanisms against HBV infection is vaccination of health care workers, which provides more than 90% protection [4]. The World health organization (WHO) has been recommended vaccination of high-risk groups like health workers, medical and health science students [11]. However, coverage of the HBV vaccine in health care providers was very low in sub-Saharan Africa, a study reported 4.6–64.4% coverage of HBV vaccine in sub-Saharan Africa [4].

Several epidemiological studies conducted in Ethiopia showed varying rates of full-dose HBV vaccination among health care workers [12–15]. The studies reported 25.6%, 12.9%, 5.4%, and 20.04% coverage of full-dose HBV vaccination among health care workers in Adama, Shashemene, Bahir Dar, and meta-analysis done in Ethiopia respectively [12–15]. However, as part of the health care providers in Ethiopia, no study has determined HBV vaccination status and associated factors among students of medicine and health science, who were on a clinical attachment. Different literatures reported 16.81%, 44.3%, 22.5%, 37% of HBV vaccine coverage among students in Cameroon [16], Uganda [17], Ghana [18], and Nepal [19] respectively. The WHO’s targeted to eliminate HBV in 2030 [20]. However, knowledge, attitude, awareness, access to screening, and vaccination of health care providers remain poor in many developing countries including Ethiopia [14]. Vaccination of health science students before starting their hospital attachments is crucial to control the disease burden, protect them from infection, and reduce the risk of nosocomial infection.

Determination of HBV vaccination coverage and its associated factors among medicine and health science students was an aid to design and implement effective intervention mechanisms such as vaccination of students before starting their clinical attachments and intervene on associated factors. Assessing the level of knowledge, attitudes, and practices (KAP) of medicine and health science students towards HBV was important to design and implement awareness creation on standard safety precautions towards the prevention of HBV infection. Planning, designing, and implementation of effective interventions were based on the available and updated information. However, there is limited information on the HBV vaccination coverage and associated factors among health science students in Ethiopia despite the increasing burden of the infection. Therefore, this study aimed to determine HBV vaccination coverage and associated factors, level of KAP towards HBV among students of medicine and health science at Wolkite University, southwest Ethiopia.

## Material and methods

### Study design, area, and period

A cross-sectional study was conducted at Wolkite University in Gurage zone, South Nation Nationality and Peoples Regional State (SNNPR), Ethiopia. The University is located in the Gubriye sub-city, 13km far away from the zonal town, Wolkite which is located 158km from the capital city of Ethiopia, Addis Ababa. The University provides teaching, research, and community service in its six colleges. College of medicine and health sciences has five departments (medicine, medical laboratory science, midwifery, nursing, and public health). In this college, there were 1,739 students, out of those 530, 271, 313, 321, and 304 were in medicine, public health officers, nursing, medical laboratory science, and midwifery department respectively. Most of the health science students received 4 years of training except for the students of medicine (6 years). The study was conducted in medicine and health sciences students from November to December 2020 in the college of medicine and health science at Wolkite University, southern Ethiopia.

### Sample size determination and sampling techniques

The sample size was determined by using a single population proportion formula [n = (Zα/2)^2^p(1-p)/d^2^] with an assumption of 95% confidence level, 5% margin of error (d), 44.3% proportion (p) of HBV vaccine coverage in medicine and health science students at Makerere University, Uganda [17]. Therefore, the sample size (n) = (1.96)^2^ 0.443(0.557)/0.05^2^ = 379. After adding a 10% non-response rate we got a final sample size of 417. In the college of medicine and health science at Wolkite University, there were 1,739 students (530 medicine, 271 public health officers, 313 nursing, 321 medical laboratory sciences, and 304 midwifery students). The total sample size (417) was distributed proportionally to each department based on their student population, accordingly, 127, 65, 75, 77, and 73 sample size was allocated to medicine, public health officers, nursing, medical laboratory science, and midwifery department, respectively. The number of study participants enrolled from each academic year was distributed proportionally based on their student population from each academic year of the departments. Finally, the study participants were selected by a random sampling technique. Based on their attendance lists of each respective department the required number of study participants from each department were selected using a lottery method. According to their lists on the attendance sheet, each study participant was represented by a slip of paper, these were put in a box and mixed, and a sample of the required size was drawn from the box.

### Study population

Source populations were all regular medical and health science students of Wolkite University, and study participants were medical and health science students who were on a clinical attachment and voluntarily participate in the study.

### Operational definition

#### Vaccination status

Was categorized into full-dose vaccination (received three or more doses of HBV vaccine), not fully vaccinated (took one or two doses of HBV vaccine), and not vaccinated (not received any dose of HBV vaccine or not sure of their vaccination status).

#### Knowledge

Was categorized into “good knowledge” if the respondents were able to answer 70% or more of knowledge questions correctly. “Poor knowledge” if the respondents were answered less than 70% of knowledge items questions correctly [7].

#### Attitude

Categorized as; positive attitude: if the respondents were able to give the correct answer for 70% or more of attitude items. Negative attitude: if the respondents answered less than 70% of attitude items [7].

#### Practice

Categorized as; good practice: when the study participants were at least able to answer 70% or more practice items correctly, poor practice: when the participants were unable to answer 70% of practice items correctly [7].

### Data collection method

A self-administered and pre-tested structured questionnaire was used to collect data about the socio-demographic characteristics of study participants (residence, age, and gender), vaccination status of HBV (full-dose vaccinated, not fully vaccinated, and not vaccinated), knowledge towards transmission and prevention of HBV infection, attitude, and practice towards prevention of HBV. The questionnaire was adopted and developed from a previous similar study [7, 21] and pre-tested in 5% (21) of study participants in Wolkite University before starting data collection and necessary amendments were made. After consenting, a questionnaire was administered to each study participant, and data were collected. Hepatitis B virus vaccination status was considered as dependent variables and age, gender, residence, department, academic years, and level of knowledge, attitudes, and practices were considered as independent variables. The variable HBV vaccination status was categorized as full-dose vaccination, not full vaccinated, and not vaccinated. However, a final analysis (associated factors) was done for full-dose vaccination status which has a binary outcome (Yes/No). We got binary vaccination status by categorizing vaccination status as full-dose vaccinated and not full-dose vaccinated. Full-dose vaccination status was considered when study participants have received three or more doses of HBV vaccine and labeled as ‘Yes’, while those study participants who had received one or two doses of HBV vaccine, and not received any dose of HBV vaccine were considered as not full vaccinated and labeled as ‘No’.

### Data analysis

After checking all questionnaires for completeness, accuracy, and clarity by the principal investigator data were entered and analyzed using SPSS version 21 statistical software (SPSS Inc., Chicago, IL). Data were summarized by using descriptive statistics like frequencies and percentages. The association between full-dose vaccination status and independent factors was determined by binary logistic regression (bivariate and multivariate) analyses. Bivariate analysis was performed for each independent variable to select candidate variables for multivariate analysis. Variables in bivariate analysis with P-value <0.25 were taken as candidates for multivariate analysis [22]. Multivariate analysis was used to identify associated factors for the full-dose vaccination status among study participants. P-value was set at <0.05 for statistical significance.

### Ethical consideration

Ethical clearance was obtained from the Research and Ethical Review Committee of Wolkite University, College of Medicine and Health Science. Permission was obtained from Wolkite University. After explaining the importance of the study, written informed consent was obtained from each study participant. Participants were informed about the objective of the study and they were assured that the confidentiality of the data will be maintained.

## Results

A total of 417 medical and health science students belonging to five departments were enrolled in the study. All of them participated in the study, giving a response rate of 100%. The mean age of the study participants was 22.7± 1.7 years ranges from 20 to 33 years. The majority of the study participants 338 (81.1%), 251 (60.2%), and 225 (54%) were in the age groups less than 23 years, males, and rural area residents, respectively (Table 1).

**Table 1:** Socio-demographic and other related characteristics of study participants at the Wolkite University, southwest Ethiopia, 2020 (n = 417).

Variables	Categories	Frequency (n)	Percentage (%)
Gender	Female	166	39.8
	Male	251	60.2
Age in years	<23 years	338	81.1
	≥23 years	79	18.9
Residence	Rural	225	54
	Urban	192	46
Academic years	Second	106	25.4
	Third	131	31.4
	Fourth	145	34.8
	Fifth	35	8.4
Department	Medicine	127	30.5
	Public health officer	65	15.6
	Nursing	75	18
	Medical Laboratory Science	77	18.5
	Midwifery	73	17.5

### Vaccination status of the study participants

Out of the 417 study participants, 24(5.8%) [95%CI: 3.8–7.9] received a full dose of the HBV vaccine in this study. The majority of the study participants 310 (74.3%) have never received the hepatitis B vaccine and 107 (25.7%) had received one or two doses of the HBV vaccine. The most frequently mentioned reasons for not being vaccinated against HBV were unavailability of the vaccine 320(81.4%), high cost of the vaccine 54(13.7%), lack of knowledge about vaccine 16(4.1%), and fear of side effects 3 (0.8%). Forgot to receive follow-up dose 216(55%), busy schedule, 47(12%), and having obtained the immunity 130(33.1%) were the common reason for not receiving a full course of vaccination among the study participants.

### Knowledge level of the study participants on HBV

The majority of the study participants 307 (73.6%) had good knowledge about HBV infection, mode of transmission, and prevention. Among the study participants, 364 (87.3%) knew that HBV infection causes liver cancer. The majority of the study participants 384(92.1%) and 400(95.9) reported contact with contaminated blood and body fluid of HBV carrier, and unsterilized syringes, needles, and surgical equipment were the main mode of transmission for HBV respectively. Regarding knowledge on vaccination, 349(83.7%) participants were aware of the HBV vaccine and it provides protection against HBV infection, and also 199(47.7%) of participants knew that there is post-exposure prophylaxis for HBV (Table 2).

**Table 2:** Knowledge level of the study participants at Wolkite University, 2020 (n = 417).

Knowledge item questions	Yes	No	Not sure
	n (%)	n (%)	n (%)
HBV causes liver cancer	364(87.3)	27(6.5)	26(6.2)
HBV carriers can transmit the infection to other	341(81.8)	62(14.9)	14(3.4)
HBV spread by casual contact such as hand shacking	299(71.7)	93(22.3)	25(6)
HBV spread by contact with open wounds or cut?	381(91.4)	17(4.1)	19(4.6)
HBV can be transmitted by contaminated blood and body fluid	384(92.1)	31(7.4)	2(0.5)
HBV can be transmitted by unsterilized syringes, needles, and surgical instruments	400(95.9)	13(3.1)	4(1)
Hepatitis B can be cured or treated	181(43.4)	156(37.4)	80(19.2)
The vaccine can prevent hepatitis B infection	349(83.7)	44(10.6)	24(5.8)
Do you think HBV has a laboratory test	266(63.8)	132(31.7)	19(4.6)
HBV has post-exposure prophylaxis	199(47.7)	184(44.1)	34(8.2)

### Attitudes of the study participants towards HBV infection

Among the study participants, 151 (36.2%) had positive attitudes towards HBV infection and risk perceptions. About 313 (75.1%) study participants agreed that following infection control guidelines would protect them from being infected at work, and 286(68.6%) were aware that the HBV vaccine is safe and effective against HBV infection (Table 3).

**Table 3:** Attitudes of study participants towards hepatitis B prevention at Wolkite University, 2020 (n = 417).

Attitude item questions	Agree	Disagree	Not sure
I have no concern about being infected with HBV	164(39.3)	235(56.4)	18(4.3)
Hepatitis B vaccine is safe and effective	286(68.6)	78(18.7)	53(12.7)
Changing gloves during blood collection and tests is a waste of time	113(27.1)	286(68.6)	18(4.3)
All patients should be tested for HBV before they receive health care	227(54.4)	164(39.3)	26(6.2)
I do not feel comfortable taking care of people with HBV	181(43.4)	230(55.2)	6(1.4)
Following infection control guidelines would protect from being infected with HBV at work	313(75.1)	95(22.8)	9(2.2)

### The practice of the study participants towards HBV prevention

Among the study participants, 196(47%) had good practice towards the prevention of HBV. Out of the 417 study participants, only 161(38.5%) had screened for HBV, 107(25.5%) had been vaccinated against HBV. However, out of the vaccinated participant, 24(5.4%) had completed the recommended three doses. About 153(36.5%) reported they had encountered needle stick injury and 278(68.8%) reported they would always change gloves for each patient during procedures (Table 4).

**Table 4:** Practices of medicine and health sciences students towards hepatitis B prevention at the Wolkite University, 2020 (n = 417).

Practice item questions		Yes	No
Have you ever screened for hepatitis B?		161(38.6)	256(61.4)
Have you got vaccinated against HBV?		107(25.7)	310(74.3)
How many doses of HBV vaccine did you receive?	One dose	39(9.4)	-
	Two dose	44(10.6)	-
	Three dose	24(5.8)	-
I always change gloves for each patient during blood taking?		287(68.8)	130(31.2)
Have you ever had a needle prick injury?		153(36.5)	263(63.1)
I always report for needle stick injury?		194(46.5)	223(53.5)

### Factors associated with full-dose vaccination status

Association between full-dose vaccination status and independent variables were tested with binary logistic regressions. By considering P-value <0.25 in bivariate analysis, gender, age, residence, academic year, department, attitude, and practice were identified candidates variables for multivariate analysis. Then, multivariate logistic regression analysis was performed to control for confounders and identify significantly associated factors with the full-dose vaccination status. After multivariate analysis, male gender, rural residence, academic years, medicine department, good attitude, and practices were significantly associated with full dose vaccination status (Table 5).

**Table 5:** Factors associated with full-dose vaccination status among study participants, 2020.

Variables	Categories	Full dose vaccination		COR(95%CI)	P-value	AOR(95%CI)	p-value
		Yes	No				
Gender	Female	15(9)	151(91)	1		1	
	Male	9(3.6)	242(96.4)	2.6(1.14–6.2)	0.024	8.8(2.9–27)	<0.001**
Residence	Rural	8(3.6)	217(96.4)	2.5(1.03–5.8)	0.042	3.6(1.2–10.6)	0.01**
	Urban	16(8.3)	176(91.7)	1		1	
Academic year	Second	5(4.7)	101(95.3)	5.9(1.8–19.7)	0.003	11.7(2.7–50.9)	0.001**
	Third	5(3.8)	126(96.2)	7.4(2.2–24.5)	0.001	19(4.25–45)	<0.001**
	Fourth	6(4.1)	139(95.9)	6.8(2.2–21.3)	0.001	27(5.8–56)	<0.001**
	Fifth	8(22.9)	27(77.1)	1		1	
Department	Medicine	4(3.1)	123(96.9)	3.7(1.09–13)	0.035	5.9(1.2–29)	0.02**
	PHO	3(4.6)	62(95.4)	2.5(0.6–10)	0.18	1.5(0.3–6.9)	0.6
	Nursing	5(6.7)	70(93.3)	1.7(0.5–5.5)	0.36	2.3(0.5–9.3)	0.2
	MLS	4(5.2)	73(94.8)	2.2(0.6–7.8)	0.2	2.8(0.6–12)	0.15
	Midwifery	8(11)	65(89)	1		1	
Attitude	Positive	15(9.9)	136(90.1)	0.3(0.13–0.7)	0.008	0.44(0.1–1.1)	0.03**
	Negative	9(3.4)	257(96.6)	1		1	
Practice	Good	19(9.7)	177(90.3)	0.2(0.07–0.5)	0.003	0.17(0.05–0.5)	0.004**
	Poor practice	5(2.3)	216(97.7)	1		1	

1; reference category,

**; significantly associated factors p-value <0.05; AOR; adjusted odds ratio, COR; crude odds ratio, MLS; medical laboratory science, PHO; public health officers.

Being male gender (AOR: 8.8; 95%CI: 2.9–27), rural residence (AOR: 3.6; 95%CI:1.2–10.6), being second year student (AOR: 11.7; 95%CI: 2.7–50.9), third year student (AOR: 19; 95%CI: 4.25–45), fourth year student (AOR: 27; 95%CI: 5.8–56), medicine department (AOR: 5.9; 95%CI: 1.2–29), positive attitude (AOR: 0.44; 95%CI: 0.1–1.1) and good practice (AOR: 0.17; 95%CI: 0.05–0.5) were significantly associated with full-dose vaccination status (Table 5).

## Discussion

The current study attempted to determine HBV vaccination coverage and associated factors, and level of KAP towards HBV among students of medicine and health science at Wolkite University, southwest Ethiopia. World Health Organization estimated 18–39% coverage of HBV vaccine among healthcare providers in developing countries and 67–79% in developed countries [20]. However, full-dose HBV vaccination coverage among medical and health science students obtained in our study was 5.8% (95%CI: 3.8–7.9), which is much lower than the WHO estimates. These warrants need to look into the vaccination status of medical and health science students before enrolling in clinical practices and vaccinate them before starting the clinical attachments. The low HBV vaccine coverage rate among the study participants might be partly attributed due to the inaccessibility and unavailability of the vaccine because it was included in the Ethiopian immunization program in 2007. Our study finding was lower than similar studies reported from Makerere University College of Health Sciences, Uganda 44.3% [17], Nepal 37% [19], Kanchipuram, India 72.5% [22], China 60% [23], and Ethiopia 20.04% [15], Adama hospital, Ethiopia 25.6% [12]. The variation in the vaccination rates might be due to differences in socioeconomic status, sample sizes, standards of health care services across the countries, and availability of the vaccine. However, our finding was consistent with previous similar studies reported from different countries [7, 14, 21, 24].

The 5.8% full-dose vaccination rate among our respondents was low, given their increased risk of contracting HBV. In this study, the most commonly reported reason for not receiving a full course of vaccination among the study participants was forgot to receive a follow-up dose (55%) and having a busy schedule (12%). This finding was in agreement with studies conducted in Gondar University Hospital, Ethiopia [25] and China [23]. However, urgent measures are required to put in place policies that will encourage the vaccination of medical and health science students.

This study revealed that 25.7% of study participants had been vaccinated at least once against HBV. This result was comparable with studies reported from different areas. For instance, a similar study conducted in Ghana reported 22.5% of participants were vaccinated against the hepatitis B virus [18]. Another study conducted in Cameroon and Hawassa reported 26.05% [16], and 30.3% [24] of study participants had been vaccinated at least once against HBV respectively. Our finding was higher than the study reported from Haramaya, Ethiopia [21], however, it was lower than the studies reported from Uganda [17] and Nepal [19]. The difference in the vaccination coverage might be due to the unavailability and inaccessibility of the vaccine in the hospitals, lack of clear policy in vaccination of HBV in health providers, and the difference in the health system of the countries.

The current study revealed that 74.3% of study participants have never received the hepatitis B vaccine. Frequently mentioned reasons for not being vaccinated against HBV among study participants were unavailability of the vaccine (81.4%) and followed by the high cost of the vaccine (13.7%). A similar finding was reported from Gondar University Hospital, Ethiopia [25], and Makerere University, Uganda [17].

Male study participants were 12% less likely to take full-dose hepatitis B vaccination than females study participant. This finding was consistent with the study conducted in Wolayita hospital in Southern Ethiopia [26], and Shashemene, Ethiopia [13], which revealed a higher vaccination rate among female participants. Other similar studies conducted in Uganda [17], and Ethiopia [15] reported similar findings. This might be because women have a higher risk perception than men, and women are more concerned about their health.

The study participants who were rural area residents were 64% less likely to receive full-dose vaccination against HBV as compared to urban residents. This might be due to a lack of awareness of the infectious disease, inaccessibility, and unavailability of the HBV vaccine. In the current study, full-dose vaccination status was significantly associated with the academic years of the study participants. This result was in agreement with a study conducted among students in Ho, Ghana [27], and Makerere University, Uganda [17]. This might be because there is better awareness of the occupational hazards and a higher acceptance rate of vaccine as their academic year increase.

Being in a medicine department was significantly associated with the full-dose vaccination status in this study. This was consistent with the studies conducted in Gondar University Hospital, Ethiopia [25] and China [23]. Study participants who had positive attitudes and good practices towards HBV were more likely to take full-dose vaccination than their counterparts. This finding was in agreement with a study conducted among health care workers at Wolayita Sodo hospital in Southern Ethiopia [26]. This might be because of better awareness and practice about the occupational hazards of HBV which makes them have a higher acceptance of vaccination than their counterparts.

Despite the different professional backgrounds of the study participants, our finding revealed that 73.6% of study participants had a good level of knowledge regarding HBV, its mode of transmission, and prevention. This finding was consistent with the previous studies reported from Makerere University, Uganda (74.6%) [17], Kochi, India (79.1%) [28], and Jimma University Medical Center, Ethiopia (73.9%) [29]. But, it was higher than studies reported from Ghana (37%) [18], Haramay University, Ethiopia (56.2%) [21], Senegal 27% [29], and Kanchipuram, India 43% [22]. The difference might be due to the variation in health policies and programs across the countries; in some countries, training and orientation on universal precautions were given to health care providers before enrolling in clinical practice, which makes them know more about occupational exposures toward contagious infections. The majority of the study participants knew that exposure to infected blood or body fluid, contaminated needles, contact with non-intact skin, or unsafe sexual contacts are risk factors for HBV infection. This result was comparable with previous studies reported from Jimma, Ethiopia [21], Cameroon [16], and Kochi, India [28].

Regarding the attitude of students towards HBV and its vaccination, 36.2% of study participants had positive attitudes regarding HBV. The majority (75.1%) of the participants were aware that following infection control guidelines will protect them from the risk of contracting HBV at the workplace and 68.6% of them believe that the HBV vaccine is effective and safe. Several previous studies reported comparable results with the current study. The study conducted in Senegal among 315 medical students reported that 32.4% of students had positive attitudes regarding HBV. Whereas, it was lower than the study finding reported from Kochi, India 84.3% [28] and Kanchipuram, India 62.5% [22]. This might be due to a difference in the sample size across the study.

In the current study, 47% of the participants had good practices against hepatitis B. This finding was higher than the study reported from Senegal 32.4% [29]. But it was lower than the study reported from Kanchipuram, India 72.5% [22]. However, our finding was in line with the study reported from Kochi, India [28], and Nepal [19]. The current study revealed that more than half of the study participants had malpractice towards HBV prevention, despite a good level of knowledge against HBV and its prevention measures. Better strategies need to be found to translate the level of knowledge into better preventive practices among the study participants. Risky practices among the study participants were highly prevalent; 36.5% of participants had been exposed to needle prick injury during their professional activities, and 46.5% had no intention to report the accident. This finding suggests that there is a need to address the barriers by strengthening training on universal safety precautions for the prevention of blood-borne pathogens.

Despite extensive efforts that have been made to minimize possible shortcomings of this study, the findings of this study were interpreted by considering the following limitations. HBV vaccination status was assessed based on self-report, we did not measure the anti-hepatitis B surface antibody titer of the participants, and did not confirm the information regarding HBV vaccination because the study participants came from different parts of the county. The cross-sectional nature of the study does not confirm a cause-and-effect relationship. There was also a possibility of admitting recall bias because of the self-reported vaccination status. Despite the limitations, the findings of this study highlighted that there was a critical need for vaccination of students against this highly contagious infection before they start clinical attachments, provisions of training on prevention mechanisms, and universal precautions regarding blood-borne pathogens before enrolling in professional practice.

## Conclusion

Our study revealed that only small proportions (5.8%) of study participants received full dose vaccination against HBV. Gender, residence, academic years, type of profession, attitude, and practices towards HBV were significantly associated factors with full dose vaccination status. Unavailability and high cost of the HBV vaccine were frequently mentioned reasons among study participants for not being vaccinated against HBV. Our finding showed that students are at a very high risk of contracting HBV infection during their clinical training owing to the low HBV vaccine uptake rate. Thus, we recommend that all students in the health care professional should be vaccinated before they enter into clinical attachments, provisions of training for students on infection prevention mechanisms, and universal precautions particularly on HBV, increase uptake of the HBV vaccine, and creating awareness on attitudes and practices of students towards HBV to enhance uptake of the vaccine.

## Supporting information

S1 File English version questionary. (DOCX)

S2 File HBV SPSS data set. (SAV)
