# Supplementary material for: Hepatitis B vaccination status and associated factors among students of medicine and health sciences in Wolkite University, Southwest Ethiopia: A cross-sectional study
Source: PLoS One. 2021 Sep 21;16(9):e0257621. doi: 10.1371/journal.pone.0257621 (PMC8454964; doi:10.1371/journal.pone.0257621)
Supplement: S1 File — (DOCX) [file pone.0257621.s001.docx]

| 1. Gender | 1. Male 2. Female |
| --- | --- |
| 2. Age in year | ________________ |
| 3.Indicate your department | 1. Medicine  2. Medical laboratory science  3. Public health officer  4. Nursing  5. Midwifery |
| 4.Residence(your background residence) | 1. Rural 2. Urban |
| 5. Academic year | ____________________ |
| **Section B Knowledge Items** | |
| 1. Can hepatitis B virus cause liver cancer? | 1. Yes 2. No 3.Don’t know |
| 2. Can HBV carriers can transmit the infection to other | 1. Yes 2. No 3. Don’t know |
| 3. Can hepatitis B be caught through casual contact such as holding of hands? | 1. Yes 2. No 3. Don’t know |
| 4. Can hepatitis B be spread through contact with open wounds/cuts? | 1. Yes 2. No 3. Don’t know |
| 5. Can Hepatitis B be transmitted by contaminated blood and blood products? | 1. Yes 2. No |
| 6. Can Hepatitis B be transmitted by un-sterilized syringes, needles and surgical instruments? | 1. Yes 2. No |
| 7. Can Hepatitis B be cured or treated | 1. Yes 2. No |
| 8. Can HBV vaccine prevent Hepatitis B? | 1. Yes 2. No 3.Don’t know |
| 9. Do you think HBV has laboratory test? | 1. Yes 2. No |
| 10. Do you think that HBV has post exposure prophylaxis? | 1. Yes 2. No |
| **Section C Attitude Item Questions** | |
| 1. I am not at risk for getting hepatitis B | 1. Agree 2. Disagree 3. Not sure |
| 2. I do not believe in the hepatitis B vaccine | 1. Agree 2. Disagree 3. Not sure |
| 3. Changing of the gloves during blood collection and test is a waste of time | 1. Agree 2. Disagree 3. Not sure |
| 4. All patients should be tested for HBV before they receive health care | 1. Agree 2. Disagree 3. Don’t have any idea. |
| 5. I do not like treating people with HBV | 1. Agree 2. Disagree 3. Don’t have any idea. |
| 6. Following infection control guidelines will protect me from being infected with HBV at work. | 1. Agree 2. Disagree 3. Don’t have any idea. |
| **Section D Practice Item Questions** | |
| 1. Have you done screening for Hepatitis B? | 1. Yes 2. No |
| 2. Have you got yourself vaccinated against Hepatitis B? | 1. Yes 2. No |
| 3. If your answer is yes in question #2, how many doses of hepatitis B vaccine did you receive? | 1. One dose  2. Two dose  3. Three dose |
| 4. If your answer is No in question #2, what was the reason for not being vaccinated? |  |
| 5. I always change gloves for each patient during blood taking? | 1. Yes 2. No |
| 6. Do you ask for a new syringe before use? | 1. Yes 2. No |
| 7. Have you ever had a needle prick injury? | 1. Yes 2. No |
| 8. I always report for needle stick injury | 1. Yes 2. No |
